# Comparing cardiorespiratory fitness and physical activity levels between third- and fifth-year medical students in a South African university

**DOI:** 10.17159/2078-516X/2024/v36i1a15245

**Published:** 2024-02-15

**Authors:** N Neophytou, G Torres, R Madoka, E Mari, MK Ndlovu, EB Bope, L Van Heerden, D Constantinou, M Phaswana

**Affiliations:** Department of Exercise Science and Sports Medicine, School of Therapeutic Sciences, Faculty of Health Sciences, University of the Witwatersrand, South Africa

**Keywords:** physical activity vital signs, cardiopulmonary fitness

## Abstract

**Background:**

Cardiovascular disease (CVD) risk factors such as sedentary behaviour, decreased physical activity (PA), and low cardiorespiratory fitness lead to an increased and accelerated risk of cardiovascular disease and mortality. Medical students tend to adopt sedentary lifestyles due to a demanding curriculum. This may have a negative effect on CVD risk factors and cardiorespiratory fitness levels of medical students.

**Objectives:**

To compare physical activity and cardiorespiratory fitness levels in a cohort of third- and fifth-year undergraduate medical students in a South African university.

**Methods:**

Data from 123 third-year and 139 fifth-year medical students in the Graduate Entry Medical Programme (GEMP) at the Faculty of Health Sciences, University of the Witwatersrand, were collected. Measurements included CVD risk factors, height, weight, blood pressure, waist circumference, cardiorespiratory fitness, physical activity vital signs and pre-participation health screening questionnaires. Descriptive statistics were presented as mean ± standard deviation or median [interquartile range] depending whether the data were normally distributed or not.

**Results:**

Both groups had low cardiorespiratory fitness when compared to norm values (GEMPI VO2 peak was 29.1 ± 5.9 ml.kg-1.min-1 and GEMPIII VO2 peak was 30.0[11.0] ml.kg-1.min-1). Most participants did not meet WHO physical activity requirements (GEMP I: 72%; GEMP III: 78%). There were significant differences in BMI (p=0.046), diastolic blood pressure (p=0.034) and VO2 peak (p=0.00001) between students meeting and not meeting WHO physical activity requirements (p<0.05).

**Conclusion:**

Third- and fifth-year medical students at a South African university fail to meet recommended WHO physical activity levels and are below cardiorespiratory fitness norms. Therefore, medical institutions should promote and implement targeted physical activity interventions to reduce the prevalence of low fitness levels and the associated health hazards among their students.

A healthy lifestyle is one of the major factors in cardiovascular disease (CVD) prevention.^[1]^ Engaging in physical activity to prevent CVD should be part of everyday life, beginning in childhood and continuing through adolescence into adulthood.^[1]^ Specifically, education regarding CVD prevention and leading a healthier lifestyle should be emphasised amongst medical students, as they are known to adopt less physically active or sedentary lifestyles throughout their studies.^[2]^

CVD risk factors, such as age, family history, smoking, obesity, hypertension, and diabetes mellitus, have been associated with CVD-related deaths.^[3]^ In addition, physical inactivity and low levels of cardiorespiratory fitness have been shown to be independent risk factors for CVD and can impact mortality.^[4]^ It is important to identify and prevent risk factors for cardiovascular disease at an early age to improve the future health of young people.^[1]^

Cardiorespiratory fitness, assessed through exercise testing, is a more precise and reliable measure than when assessed through self-reported physical activity levels.^[5]^ The latter has primarily been used in research on CVD risk, health status, and lifestyle factors of medical students.^[6–8]^

Torres et al.^[9]^ found that physical activity and cardiorespiratory fitness levels were sub-optimal (low) in fifth-year medical students at a South African university. It is not known whether younger medical students share this characteristic. This study aimed to compare third- and fifth-year medical students at the same university to determine changes across the two years of study.^[9]^ These insights are important, as the students represent future medical doctors who may have to practice physical activity behaviour and lifestyle modification counselling with their patients.

## Methods

### Study design

A cross-sectional study design was utilised. All registered third-year (GEMP I) and fifth-year (GEMP III) medical students in the Graduate Entry Medical Programme at the Faculty of Health Sciences, University of the Witwatersrand, were invited to participate in the study. Data were collected during a practical teaching session that involved the measurement of CVD risk factors and cardiorespiratory fitness. Eligible participants were informed that their data may be used for research analysis. Before participation, written informed consent was obtained. Students who did not attend the clinical session or did not want their data to be used did not have their data recorded. Furthermore, students could withdraw consent of data at any point without repercussion.

### Ethical approval

This study was granted ethical clearance by the Human Research Committee of the University of the Witwatersrand (clearance number: M200349).

### Participants

A sample of convenience was used for this study. All students who were registered in the GEMP I and GEMP III programmes (equivalent to third- and fifth-year medical students, respectively) were included in the study. A total of 123 GEMP I students and 139 GEMP III students participated in the study. Any students who were diagnosed with CVD, such as myocardial infarction, heart failure, heart transplantation, congenital heart disease, or stroke, were not included in the study. Also, students who did not give informed consent were excluded. We included measurements from students who did not have a complete data set and reported total numbers (n) for each variable to reflect this inclusion, as the variables were mutually independent. Only complete data sets were used for the comparative group analysis of students who met and did not meet WHO physical activity recommendations.

### Testing procedures

Testing procedures for Physical Activity Vital Sign (PAVS), blood pressure, height, weight, waist circumference, and the cardiorespiratory fitness level followed the same methodology and equipment as described by Torres et al. ^[9]^

### Statistical analysis

Descriptive statistics are presented as mean ± standard deviation or median (interquartile range). Data are expressed as median and interquartile range for data not normally distributed or mean and standard deviation for normally distributed data. Note that the same variable may have been expressed in both statistical formats since two unpaired cohorts were analysed. Cardiorespiratory fitness, physical activity levels, and anthropometric measurements (weight (kg), waist (cm), height (m)) were compared between the GEMP I and GEMP III medical student groups respectively. In addition, a Chi-square test was used to compare the group of students meeting the WHO physical activity recommendations to those who did not meet the criteria. Effect size calculations were conducted, and <0.2 was considered trivial, 0.2 to 0.5 was considered *small*, 0.5 to 0.8 was considered *medium* and >0.8 was considered a *large* effect. T-tests were used to compare the normally distributed data and Mann–Whitney U tests were used for non-parametric analysis. All statistical analysis was conducted using Statistica version 13.2 (Statistica, Tulsa, USA).

## Results

Table 1 compares the physical and physiological characteristics of the medical student groups. Seventy-one per cent (71%) and sixty-five per cent (65%) of the participants were female in the GEMP I and GEMP III groups, respectively. The results indicated that both cohorts of students had physical and physiological values in the healthy ranges, except for cardiorespiratory fitness (VO_2 peak_) which was below the reference ranges for the median age group (Table 1). In addition, no significant differences were noted between the physical and physiological characteristics of the two groups (except for age, which was expected) (Table 1). Mann-Whitney U tests showed a significant difference in age between the GEMP I and III groups (U = 3824, p =0.001). No other significant difference was identified between the two groups.

One hundred and six (n=106) and one hundred and forty-eight (n=148) of the students in GEMP I and III, respectively, fully completed the PAVS questionnaire. Both groups of medical students yielded a high percentage of physical inactivity. The majority of the GEMP I (n= 76) (72%), and GEMP III (n=115) (78%) students did not meet the WHO physical activity requirements. A Chi-square test showed no significant difference in the percentage of students not meeting the WHO physical activity requirements between the 3rd- and 5th-year groups (p=0.275).

Table 2 depicts the combined results of the GEMP I and GEMP III groups, comparing those meeting the WHO physical activity recommendations with those not meeting the recommendations. T-tests and Mann–Whitney U tests showed significant differences in BMI (p=0.046), diastolic blood pressure (p=0.034), and VO_2_ peak (p=0.00001) parameters between students who met the WHO physical activity requirements and those who did not meet the WHO requirements (Table 2). Those who met the physical activity requirements fared better than those who did not meet the guidelines.

## Discussion

This study compared physical activity levels, anthropometric measures and cardiorespiratory fitness between two cohorts of undergraduate medical students, two years apart for their education curriculum (third- and fifth-year of a 6-year degree). Students in both cohorts had physical and physiological values in the healthy ranges, except for cardiorespiratory fitness (VO_2_ peak), which was below the reference ranges for the median age group (Table 1). Only age was significantly different between the two cohorts, as expected. All other parameters were similar in the two cohorts, which indicates that medical students did not change cardiorespiratory fitness or physical activity levels as they advanced through their studies.

The WHO recommends 150 minutes per week of moderate physical activity or 75 minutes per week of vigorous physical activity. In addition, muscle strengthening for two days of the week is recommended.^[11]^ An important finding of this study is that most 3rd- and 5th-year South African students at this university did not meet the global physical activity levels. Kunene and Taukobong reported similar findings in healthcare workers, where 29% participated in moderate physical activity, while 40% presented with low physical activity levels per week.^[12]^

A study by Prioreschi et al. reported that the majority of young South African adults spent a concerning amount of time engaging in sedentary behaviour and did not show accumulating activity in prolonged bouts.^[13]^ These findings agree with this study’s results, as the medical students had healthy characteristics but were unfit, with low physical activity levels. Furthermore, a study by Gresse et al. also found that health science university students (n=619) at another South African university (Nelson Mandela University) had reduced physical activity levels and were not meeting WHO guidelines.^[14]^ Similar to our study, Penpid et al. found that university students presented with high levels of physical inactivity.^[15]^

Contrary to our findings, several studies have reported a significant proportion of medical students meeting WHO physical activity guidelines, particularly in developed countries. Interestingly, Grujiči et al. reported that regular physical activity engagement is directly linked with higher socioeconomic status. Their study also found a gender discrepancy, whereby males were more physically active than their female counterparts.^[16]^ Consistent with our findings, a study investigating CVD risk factors and the cardiorespiratory fitness levels of South African fifth-year medical students,^[9]^ demonstrated lower levels of physical activity and cardiorespiratory fitness than recommended for health and wellbeing.

In addition, our study found that medical students consistently presented with low physical activity and cardiorespiratory fitness levels despite the year of study. The finding may also be attributed to socioeconomic status, but this must be confirmed. In this sample specifically, an additional factor to consider would be their medical education regarding physical activity guidelines and fitness norms. Implementing physical activity interventions early in the degree programme could prevent poor health trends.

Low- and Middle-Income Countries (LMIC), including South Africa, are undergoing a rapid transition to urbanisation, which poses a negative impact on lifestyles, such as physical inactivity, poor dietary habits, and sleeping patterns.^[17]^ Medical students have also been affected by urbanisation and a struggle to lead a healthy lifestyle due to increased demands of academic responsibilities and expectations.^[18,19]^ Furthermore, Belfrage et al. emphasised that the health and lifestyle practices of future medical doctors might influence their patient counselling and interaction abilities.^[20]^

Although the majority of participants in this study presented with BMI and blood pressure values within the normal range (for both the GEMP I and III groups respectively), this study showed significant differences in BMI (Body Mass Index) (p=0.046), diastolic blood pressure (p=0.034), as well as VO_2_ peak (p=0.00001) parameters between students that either met or did not meet the WHO physical activity recommendations. Implementing physical activity interventions early in the degree program could prevent poor health trends.

The high level of physical inactivity among the medical students most likely contributed to the below-average cardiorespiratory fitness levels, as measured by VO_2_ peak evident in both cohorts (Table 1). Improving cardiorespiratory fitness may be important among medical students, as it has been shown to independently predict CVD and all-cause mortality.^[17, 21]^

### Limitations

Our study had some limitations, for example, the cross-sectional study design was limited to using self-reported instruments for physical activity levels. Some students from both groups chose not to record or perform some measures and, as such, there are incomplete records. Therefore, there is a need for further investigations involving a larger sample and using objective tools to measure physical activity.

## Conclusion

A healthy lifestyle is one of the vital keys to combating cardiovascular diseases and mental well-being in young adults. This emphasises the importance of promoting a healthy and active lifestyle among medical students, who tend to adopt sedentary habits while studying. This study found that a high percentage of 3rd and 5th year South African medical students did not meet the physical activity levels recommended by the WHO and had below-average cardiorespiratory fitness levels. This is concerning not only for their personal health but also for promoting physical activity as a way to prevent and treat chronic lifestyle diseases. Intervention programmes should be developed to increase physical activity and cardiorespiratory levels in medical students.

## Figures and Tables

**Table 1:** Comparison of physical and physiological characteristics of the 3^rd^ (n=123) and 5^th^ (n=139) year medical student groups

Parameter	GEMP I		GEMP III		P-value

	n	Value	n	Value	

**Age (y)**	123	22.0 [2.0]	139	23.0 [1.0]	0.001*
**Weight (kg)**	113	61.1 [19.5]	139	64.7 [17.6]	0.413
**Height (m)**	113	1.6 [0.1]	138	1.7 [0.1]	0.408
**Waist circumference (cm)**	106	74.3 [17.0]	134	76.0 [16.5]	0.822
**Body Mass Index (kg.m** **^−2^** **)**	113	22.8 [6.4]	138	22.9 [5.1]	0.718
**Systolic blood pressure (mmHg)**	113	119.0 ± 12.6	135	118.0 [12.0]	0.118
**Diastolic blood pressure (mmHg)**	113	78.0 [15.0]	134	78.0 [11.0]	0.754
**VO** **_2_** ** peak (ml.min** **^−1^** **.kg** **^−1^** **)**	41	29.1 ± 5.9**	81	30.0 [11.0]**	0.567

Data are expressed as median [interquartile range] for data not normally distributed or mean ± standard deviation for normally distributed data.

* indicates p < 0.05;

** indicates VO_2_ peak data outside of reference ranges.^[10]^ Reference values for age group (20–29 years): Men: 44 mlO_2_.kg^−1^.min^−1^ and Women: 31.6 mlO_2_.kg^−1^.min^−1^. Subjects were included if they did not have a complete data set, the reported total numbers (n) for each variable reflect this inclusion. VO_2_ peak, peak oxygen uptake; GEMP I, 3^rd^ year students; GEMP III, 5^th^ year students.

**Table 2:** Comparison of students who were meeting WHO physical activity requirements to those who were not meeting the requirements (combined GEMP I and GEMP III groups)

Variables	Students not meeting the WHO requirements		Students meeting the WHO requirements		P-value	Effect size (*effect category*) (95% confidence interval)

	n	Value	n	Value		

**Weight (kg)**	103	67.2 ± 16.0	32	63.4 ± 12.7	0.111	−0.25† (*small)* (−0.65–0.15)
**Waist circumference (cm)**	99	77.0 ± 14.5	31	74.7 ± 9.2	0.205	−0.17 (*trivial)* (−0.56–0.23)
**Body Mass Index (kg.m** **^−2^** **)**	103	23.4 [5.4]	32	22.2 [4.2]	0.046*	−0.23† (*small)* (−0.63–0.16)
**Systolic blood pressure (mmHg)**	100	117.6 ± 8.6	32	115.8 ± 8.0	0.142	−0.21† (*small)* (−0.61–0.19)
**Diastolic blood pressure (mmHg)**	100	77.0 ± 10.6	31	72.8 ± 12.7	0.034*	−0.38† (*small)* (−0.78–0.03)
**VO** **_2_** ** peak (ml.min** **^−1^** **.kg** **^−1^** **)**	55	27.9 ± 7.4	24	37.3 ± 12.5	0.000*	1.02† (*large)* (0.51–1.52)

Data are expressed as median [interquartile range] for data not normally distributed or mean ± standard deviation for normally distributed data.

* indicates p < 0.05;

† indicates effect size >0.2.

WHO physical activity requirements: adults should do at least 150–300 min of moderate-intensity aerobic physical activity, or at least 75–150 min of vigorous-intensity aerobic physical activity, or an equivalent combination of moderate-intensity and vigorous-intensity activity throughout the week and should also do muscle-strengthening activities at moderate or greater intensity that involve all major muscle groups on 2 or more days a week ^[111^ Subjects were included if they did not have a complete data set, the reported total numbers (n) for each variable reflect this inclusion. VO_2_ peak, peak oxygen uptake; WHO, World Health Organisation
